# Identification of the NADH-oxidase gene in *Trichomonas vaginalis*

**DOI:** 10.1007/s00436-019-06572-8

**Published:** 2019-12-18

**Authors:** Aline Lamien-Meda, David Leitsch

**Affiliations:** Institute for Specific Prophylaxis and Tropical Medicine Center for Pathophysiology, Infectiology, and Immunology, Medical University of Vienna, Kinderspitalgasse 15, A-1090 Vienna, Austria

**Keywords:** Trichomonas vaginalis, Anaerobiosis, Oxygen scavenging, NADH oxidase

## Abstract

The microaerophilic human parasite *Trichomonas vaginalis* causes infections in the urogenital tract and is one of the most often sexually transmitted pathogens worldwide. Due to its anaerobic metabolism, it has to quickly remove intracellular oxygen in order to avoid deactivation of essential metabolic enzymes such as oxygen-sensitive pyruvate:ferredoxin oxidoreductase (PFOR). Two major enzyme activities which are responsible for the removal, i.e. reduction, of molecular oxygen have been identified in *T*. *vaginalis* flavin reductase, formerly designated NADPH oxidase, which indirectly reduces oxygen to hydrogen peroxide via flavin mononucleotide (FMN), and NADH oxidase which reduces oxygen to water. Flavin reductase has been identified and characterized at the gene level as well as enzymatically, but NADH oxidase has so far only been characterized enzymatically with enzyme isolated from *T*. *vaginalis* cell extracts. In this study, we identified NADH oxidase by mass spectrometry after isolation of the enzyme from gel bands positively staining for NADH oxidase activity. In strain C1 (ATCC 30001) which is known to lack NADH oxidase activity completely, the NADH oxidase gene has a deletion at position 1540 of the open reading frame leading to a frame shift and, as a consequence, to premature termination of the encoded polypeptide.

**Electronic supplementary material:**

The online version of this article (10.1007/s00436-019-06572-8) contains supplementary material, which is available to authorized users.

## Introduction

The microaerophilic parasite *Trichomonas vaginalis* is one of the most prevalent sexually transmitted pathogens worldwide, causing hundreds of millions of infections every year (Leitsch, 2016). The parasite colonizes the urogenital tract and causes urethritis, vaginitis and cervicitis. Moreover, *T*. *vaginalis* can cause adverse pregnancy outcomes (Silver et al., 2014) and facilitates infection with HIV (Masha et al., 2019). Due to its anaerobic metabolism, *T*. *vaginalis* has to effectively remove intracellular oxygen in order to prevent the deactivation of essential enzymes such as pyruvate:ferredoxin oxidoreductase (PFOR) and hydrogenase (Lloyd and Kristensen, 1985). Two major oxygen scavenging activities have been identified which both reside in the cytoplasm (Tanabe 1979; Linstead and Bradley, 1988): NADPH oxidase and NADH oxidase. The former reduces flavin mononucleotide (FMN) and other flavins through oxidation of NADPH (Linstead and Bradley 1988; Leitsch et al., 2014). Reduced FMN, in turn, rapidly reacts with molecular oxygen to form hydrogen peroxide (Chapman et al., 1999; Leitsch et al., 2014). The latter reduces molecular oxygen to water (Tanabe, 1979) by harnessing electrons derived from NADH. The gene for NADPH oxidase, which has been renamed to flavin reductase, was recently identified and characterized (Leitsch et al., 2014). Importantly, flavin reductase activity is decreased or absent in metronidazole-resistant *T*. *vaginalis* isolates (Leitsch et al., 2012, 2014). This leads to elevated intracellular oxygen concentrations and, as a consequence, to a decreased reduction of metronidazole, a prerequisite for metronidazole toxicity (reviewed in Leitsch, 2019). In contrast to flavin reductase, NADH oxidase has so far only been characterized at the protein level using enzyme isolated from *T*. *vaginalis* extracts (Linstead and Bradley, 1988). It was described to be monomeric and 98 kDa in size and to be highly vulnerable to atmospheric oxygen concentrations and to hydrogen peroxide. Recently, it was also shown that NADH oxidase activity is sharply decreased in metronidazole-treated *T*. *vaginalis* (Leitsch et al., 2014), rendering flavin reductase the only active oxygen scavenging enzyme active in the presence of oxygen. This might explain the irrelevance of NADH oxidase for metronidazole resistance (Rasoloson et al., 2001). Interestingly, one well-studied *T*. *vaginalis* strain C1 (ATCC 30001) is devoid of NADH oxidase activity (Müller and Gorrell, 1983), indicating that NADH oxidase is not strictly essential for survival of the parasite in vivo.

The aim of this study was to identify the NADH oxidase gene in order to facilitate further, more directed research on this unusual enzyme.

## Materials and methods

### Chemicals

Nitroblue tetrazolium (NBT) was purchased from Sigma.

### Cell culture

*Trichomonas vaginalis* strains C1 (ATCC 30001) and G3 (PRA-98) were grown in trypticase, yeast extract, maltose medium (TYM) (Diamond, 1957) and sub-cultured every day or every second day.

### Visualization of NADH oxidase activity by in-gel nitroblue tetrazolium blue (NBT) staining

*Trichomonas* cell extracts were prepared, and native gel electrophoresis was performed, respectively, as described (Leitsch et al., 2014). Briefly, after harvest of cells and washing of cells in 1 × PBS (800×g, 5 min), cells were taken up in 100 mM Tris pH 7.5 buffer and lysed in a Dounce homogenizer. Insoluble components were removed by centrifuging at 20,000× g for 10 min (4 °C). Resulting supernatants were loaded on native polyacrylamide gels lacking SDS. NBT staining was also performed as described before (Leitsch et al., 2014), but the assay buffer in which the gels were immersed for staining contained NADH instead of NADPH (100 mM Tris pH 7.5, 0.5 mM NAPH, and 0.2% NBT). After staining, gels were scanned and evaluated.

### Mass spectrometry of excised bands

The upper gel band was excised and analysed as described (Leitsch et al., 2014). Briefly, proteins in gel bands were digested with trypsin and subsequently analysed by reversed phase LC-ESI ion trap tandem mass spectrometry using an UltiMate 3000 UHPLC system (Dionex, part of Thermo Fisher) coupled to an amaZon speed ETD ion trap (Bruker Daltonics, Bremen, Germany). MS/MS spectra were analysed with Data Analysis 4.0 (Bruker Daltonics) and searched against the NCBI protein database (Version 28/08/2011, containing 15,148,518 sequences) using the in-house Mascot Server 2.3 (Matrix Science, UK) and ProteinScape 3.1 (Bruker Daltonics, Bremen, Germany). The same search parameters as given before (Leitsch et al., 2014) were applied. All analyses were performed in duplicate.

### Cloning of the NADH oxidase gene into the pET-17b vector

The NADH oxidase gene was amplified by PCR from genomic DNA derived from *T*. *vaginalis* G3 cells using the following primers: TACGTACGCATATGCTTAAAATACAGCAGCTCACTGAAGA (forward) and TCATCCAGGGTACCTTAGTGATGGTGATGGTGATGGAAGATTTCAGCCATGATGC (reverse). The forward primer contains a flanking NdeI restriction site whereas the reverse primer encodes a 6× histidine tag for protein purification which was preceded by a KpnI restriction site. The PCR product was digested with NdeI and KpnI and ligated into the pET-17b vector.

### Expression of NADH oxidase in *Escherichia coli* BL21-AI

The pET vector carrying the NADH oxidase gene was transformed into *E. coli* BL21-AI™ (Thermo Fisher Scientific) for expression of NADH oxidase. Protein expression was started in 50 ml batch cultures (OD = 0.4–0.6) at 37 °C upon addition of L-arabinose (0.1%) and conducted without shaking to keep oxygen levels low. After expression, bacteria were lysed by grinding in a chilled mortar (− 20 °C) with a pestle. Lysates were centrifuged at 20,000× g at 4 °C to remove cell debris and chromosomal DNA, and resulting supernatants were loaded on Ni-NTA agarose columns (Qiagen). Recombinant NADH oxidase was purified from the extract via its 6× histidine tag.

### Sequencing of the NADH oxidase gene from strain C1

Five overlapping fragments spanning the whole NADH oxidase gene were amplified from genomic DNA from strain C1 (for primers see Supplementary Table 1) and submitted for sequencing to Eurofins Genomics. This procedure was repeated once in order to ensure reproducibility.

## Results and discussion

**Fig. 1:**
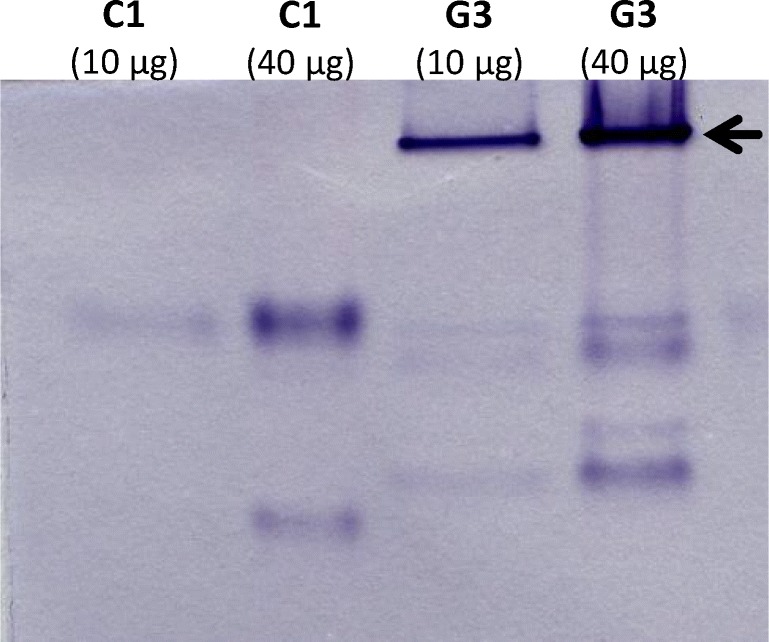
In-gel staining with extracts of G3 and C1 after native PAGE using NADH. The upper band is completely missing in C1 which is known to lack NADH oxidase activity

In analogy with previous work on flavin reductase (Leitsch et al., 2014), identification of NADH oxidase was attempted in native polyacrylamide gels upon staining with nitroblue tetrazolium (NBT). NBT is a yellowish tetrazolium compound forming blue formazan precipitates in gels and on membranes upon reduction, thereby allowing the identification of reductases in situ. Cell extracts of trophozoites from strain C1 and G3 were loaded, and gels were stained after gel electrophoresis using NADH as electron donor. Strain G3 was chosen because it is the genome strain (Carlton et al., 2007) whereas strain C1 is known to lack NADH oxidase activity, facilitating the identification of the enzyme through comparison of the staining patterns of G3 and C1. Indeed, G3 displayed a very strong and distinct stain in the upper part of the gel which was completely absent in C1 (Fig. 1). The band was excised and analysed by mass spectrometry. Only two proteins were found in the band to have a Mascot score above 90 (indicating 95% confidence level) in two separate analyses: actin (XP_001301717 or TVAG_200190) and pyridine nucleotide-disulphide oxidoreductase family protein (XP_001315422 or TVAG_049830). Actin is one of the most abundant *T*. *vaginalis* proteins as visible on 2D-gels of *T*. *vaginalis* protein extracts (unpublished data) and therefore a likely contaminant across the gel. The second protein, however, has practically identical features to NADH oxidase as determined experimentally earlier, i.e. a theoretical size of 94,996 Da (871 aa) and a pI of 5.75 as compared to approximately 98 kDa and a pI of 5.2 (Linstead and Bradley, 1988). According to the NCBI database, the protein contains flavodoxin-like domain (aa 260–402), a rubredoxin-like domain (aa 436–469) and a pyridine nucleotide-disulphide oxidoreductase domain (aa 483–772) spanning a Rossmann fold typical of NAD(P)(+)–binding proteins (aa 485–571). This structure is fully compatible with earlier observations on NADH oxidase, i.e. oxidation of NADH and reduction of oxygen (Tanabe 1979; Linstead and Bradley, 1988). The enzyme is a flavodiiron protein (FDP) which is a class of enzymes described to occur mainly in anaerobic organisms and to reduce oxygen and/or nitric oxide (Folgosa et al., 2018).

It was attempted to express the candidate NADH oxidase in *E. coli*. The recombinant enzyme could indeed be expressed in full length (Supplementary Fig. 1) but proved to be inactive, possibly to due to prolonged exposure to oxygen levels which exceed the enzyme’s capacity. It is also possible that *E. coli* does not have the necessary capability to synthesize functional rubredoxin-containing proteins in large quantities. In order to obtain additional evidence that TVAG_049830 is indeed identical with NADH oxidase, the gene was amplified and sequenced from genomic DNA of strain C1 which lacks NADH oxidase. The gene was found to have a deletion at position 1540 (Supplementary Fig. 2) leading to a frame shift after aa 513. Premature termination of the polypeptide occurs 173 aa downstream of the frameshift mutation at position aa 684. The frameshift mutation disrupts the NAD(P)(+)–binding domain (aa 485–571), most likely leading to inactivation of the enzyme. We, consequently, conclude TVAG_049830 is identical with NADH oxidase: (1) this enzyme is prominently present in the upper band in NBT in-gel stains when using extracts from G3 but absent when using extracts from C1 (Fig. 1), a strain which lacks NADH oxidase activity. (2) The particular structure of the gene regarding size, pI and domain composition is fully compatible with earlier observations (Linstead and Bradley, 1988), and (3) the gene is mutated in strain C1 leading to an inactive enzyme.

When performing a BLAST search with the TVAG_049830 protein sequence (XP_001315422), the enzyme displays 100% identity with “A-type flavoprotein” (CAI11388) and extensive identity with two additional proteins in *T*. *vaginalis* (Table 1). It also displays high similarity with a homologue in *Tritrichomonas foetus* (Table 1). TVAG_049830 was designated as “A-type flavoprotein” (a synonym for flavodiiron protein) in a study on lateral gene transfer from bacteria to trichomonads (Andersson et al., 2006). Indeed, the closest homologue of NADH oxidase outside the trichomonadids is a protein of almost identical size in *Turicibacter sanguinis*, a genus of intestinal bacteria in the phylum Firmicutes (Table 1). It is presently unclear if the two additional NADH oxidase homologues in *T*. *vaginalis* have a physiological role. It is important to note, however, that neither was identified in the upper band of the NBT in-gel stains which suggests that the homologues are either not expressed or not active (or have different substrate specificity, respectively). Similar observations were made with flavin reductase (Leitsch et al., 2014) which has six further homologues in the genome of which only two show appreciable activity but with far lower affinity for FMN. In contrast, only one homologue of NADH oxidase exists in *T*. *foetus* (OHT07162). Thus, it is highly probable that NADH oxidase activity in *T*. *foetus* (Cerkasovová and Cerkasov, 1974) is exerted by this enzyme. Still, experimental evidence analogous to that as presented herein is needed for confirmation, because NADH oxidase activity can also be exerted by enzymes not related to TVAG_049830, such as NADH oxidase in *Giardia lamblia* (Brown et al., 1996). Finally, as soon as further trichomonadid genomes have been published, the occurrence of NADH oxidase amongst trichomonadids can be assessed more comprehensively.

**Table 1:** Sequence comparison of NADH oxidase (XP_001315422) using BLAST on the NCBI data base

Entry	Name	Organism	% identity to XP_001315422	% similarity to XP_001315422	Size (aa)
CAI11388	A-type flavoprotein, partial	Trichomonas vaginalis	100	100	852
XP_001317833	Pyridine nucleotide-disulphide oxidoreductase family protein	Trichomonas vaginalis	80	90	871
XP_001322980	Pyridine nucleotide-disulphide oxidoreductase family protein	Trichomonas vaginalis	79	89	871
OHT07162	Pyridine nucleotide-disulphide oxidoreductase family protein	Tritrichomonas foetus	55	71	910
WP_147633467	MBL fold metallo-hydrolase	Turicibacter sanguinis	52	69	870

## Electronic supplementary material

ESM 1 (PDF 186 kb)

ESM 2 (PDF 513 kb)

ESM 3 (XLSX 11 kb)
