# Supplementary material for: Identification of the NADH-oxidase gene in Trichomonas vaginalis
Source: Parasitol Res. 2019 Dec 18;119(2):683–6. doi: 10.1007/s00436-019-06572-8 (PMC6985181; doi:10.1007/s00436-019-06572-8)
Supplement: Supplementary file 1 — (PDF 186 kb) [file 436_2019_6572_MOESM1_ESM.pdf]

Supplementary figure 1:

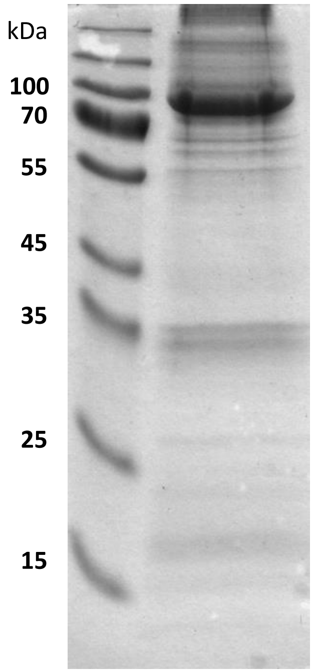

NADH oxidase was expressed in BL21-AI and isolated in NiNTA agarose columns via its 6 x His tag. The protein was eluted in 100 mM sodium phosphate buffer containing 500 mM Imidazole. Size marker: PageRuler (ThermoFisher)
