# Supplementary material for: Identification of the NADH-oxidase gene in Trichomonas vaginalis
Source: Parasitol Res. 2019 Dec 18;119(2):683–6. doi: 10.1007/s00436-019-06572-8 (PMC6985181; doi:10.1007/s00436-019-06572-8)
Supplement: Supplementary file 2 — (PDF 513 kb) [file 436_2019_6572_MOESM2_ESM.pdf]

Trichomonas vaginalis Flavoprotein C1 sequence (2835) :

ATGCTTAAAATACAGCAGCTCACTGAAGACATCTATTGGCTTGGCGTCCTTGATTCCAACCTTGCTGTCTTCGAT
ATCATCATGGAGACAAAGTATGGTACAACATACAACGCTTATCTTATAAAGACCCCAGAAGGTGCAGTTCTCGT
CGAAACAGTTAAGGAGACATTCTTCGATGAATACATCGAGAAGGTTAAGTCCGTCATCGGCGACATCCACAAG
ATCAAATACCTTATCACAAACCATACAGAACCAGATCACTCTGGCTCCATCAAGAAGATGATCGAGCTCATCCC
AGATCTCACAGTCGTTGGCTCCAAGACAGCCCTCACATACCTTGAGGATATCGTCAACATCCCATTCAAGGGCC
ACTCCGCTGAAGATCTCAAGGTCCTTAAGTTCGGTGGCAAGTCATTCGAATTCATTTCCTGCCCATTCCTTCACT
GGCCAGACTCAATGTACACATGGGTTCCAGAAGAGAAAACACTCTTCACATGCGACTCATTCGGTGCTCACTAC
TCACCAAAGAAGTCCATCCTCATGAGCCAGCTCCCACCAGAGGAAGAGGAAGGCTACCAAGATGCTCTCCTCT
ACTATTACACCGCCATTTTCGGCCCATTCAAGGAGTACGTCATCAAGGGAACAGACAAGATCCTCAACCTTGAC
ATCAAGCTCGTCGGTCTTGGCCACGGCCCAGTCCTCGATGCTCGTATCAAGGAGACAATCGATACATACCGCA
AATGGTCTGCCCCACTCCCAACACACGAAGGCAAGGAAGTCGTCATGGTCTACGCCTCTGCTTACGGCTACAC
AACAGAGATGGCCGAAGAAATCAAGGCAGGCATCCTCGCTAAGATCCCAGATGCCAAGATCAAGATGTTCAA
CGTCAACATCCAGAACTATGGCGGCCTCAAGGGCGAGATCATGAACGCAATCGCTACAGCTGACGGTGTTCTC
CTCGGCACAAACACAATCAACGGCGACGCTGTTCCACCAGTTTGGGATGTTGCCCTCTCCATGAACCCAATCGT
CCATGGCGGCAAGATCGTCACAGCATTCGGCTCATACGGCTGGTCCGGCGAAGGTGTCGACAACATCATTGCC
AGATTCGATCAGATCCGTTGCAAGGTTATCGACGGCTGCAAGATCAAGTTCCGCATGTCAAAGAAGGAACACG
GGAAGGTCAACGAATTCGGCCAGCAGTTCGGTGAAGCCCTCATCACAGGCAAGGTCCCAGAACGCGCTGTTC
CAGGCAAGGTTGTTGGTGCTAAGGACTGGTCCGAGCTCAACCCAACAGGCGCCGTCGTTCTCTGGCGTTGCGT
CATCTGCGGCGAAATCTACGCCGGTGTTACACCACCACTCCAGTGCCCAGCTTGCGGTGTCGGCCAAGACCTCT
TCGAACTCTACGAGGCCGAGCAGGTCACACACAAGTCAACAGAGCCACTCAACTACGTCATCGTTGGCTCTGG
TGCCGCCGCTGTTGCTTCCATCGAAGCCATCCGCGCTCGCAATGCCGTCGCTAAGATCACAATGATCAGC-
GTGAAAAGGTCATGCCATACTACAGACCAATCATCGTCGATGCCCTCAACGCTGAGATCGCTCCAGACAAATA
CTTCCTCAAGAACGAAGAGTGGTACAAGGAGAACAAGATCGAAATCAAGCTTGACACATCCGTCACAGCCATC
GACACAAAGGCCAAGAAGGTCAAGCTCTGCTGCGGTGATGAACTCGCCTACGACAAGCTCATCCTCGCTACAG
GTTCCCGCCCATTCATCCCACCAATCGGCCACGAGGGACTCTCCGGCGTCATCGTCATCCGTACATCCGCTGAT
GTCGACCAGCTCAAGGCCGCCTGCCAGACAGCTAAGAAGGCCGTCGTTATCGGTGGTGGTGTCCTCGGTCTCG
AGAACGCATCCGCTATCAAGGAGAAGGGCGTTGCTGTCACAGTCGTCGAGTGCATGCCACGTCTCATGGCCCG
CCAGCTCGATACAGAAGCTTCCGCTGTCCTCCTCGAGGAAGTCAAGAAGTTTGGTGTCGATGTCCGCCTCGGC
ATGACAGTTTCCATCAAGGGCGATGGCAAGCACGTCACAGGCGTCCAGGTCGGCGAGGAGTTCATCCCAGCC
GATTTCGTTGTTGTCAACGCCGGTGTCCGCGCCGAATCCGAAGTAGCCGCTGCAGCTGGCATCAAGTGCGGCC
GCGGTATCATTGTCAACAACAAGATGGAGACAAACGTTCCAGATGTCTACGCAGCTGGTGATTGCGCATTCCT
TGACAACATGAACCAGGGTCTCTGGGCTCCAGCTCTTGCTATGGGTAAGGTCGCCGGTGCTAACGCTTGCGGC
GATAACAAGACATTCGCTTTCGCTATCGAACCAGTCTCAATGATCGCTATGGGAACAGATCTCTTCGCTTGCGG
CAACCCACCAGAGAGCGCACAGGGCTACAACGTTGTCTCTCAGAAGGATGACAAGGAAGGATCCGCTATCAA
GCTCTACTTCAAGGATAATCGCCTCGTTTACGCAGCTGGCTTCAGAATCCAGAAGATCTCCGGCTCCCTCTTGA
AGGGCGTCAGAGAGGGCCGCTCATTAGAGCGCATCATGGCTGAAATCTTCTAAAGAAGATTTCCAAAAAATAA
TTTTAATAATTTCGATGTTCGAAACGGCTCGCTTTGGTAAAAAAAGTTTTAATAATCTTCTCAATTTTTTTCCAAA
TATTTTTTTCCATTGAAAAGAATTTTAAAATTGAATTAATAAAACAGTTTTCTTGATATAAATTTTGACATTCATT
TTTCAAAAATGGAAGAGTTTATTGAGCGTTTAAAGTCAATTTATGAG

Report: XM_0011315387.1 (2700)

Sequence length: 2835

Mutation positions:

483;

933 (A/C)

1506 (T/G)

1540 (-/C) deletion

1717 (T/C)

All mutation with exception of deletion are sense mutations.

Deletion position:

1540 (-/C) deletion

Section 1

```
                    1         10        20        30        40        50        60        70          82
» Contig 1        (1) ATGCTTAAAATACAGCAGCTCACTGAAGACATCTATTGGCTTGGCGTCCTTGATTCCAACCTTGCTGTCTTCGATATCATCA
» AJ864551.1       (1)                                                          AACCTTGCTGTCTTCGATATCATCA
» XM_001315387.1  (1) ATGCTTAAAATACAGCAGCTCACTGAAGACATCTATTGGCTTGGCGTCCTTGATTCCAACCTTGCTGTCTTCGATATCATCA
Contig 1          (1) ATGCTTAAAATACAGCAGCTCACTGAAGACATCTATTGGCTTGGCGTCCTTGATTCCAACCTTGCTGTCTTCGATATCATCA
```

Section 2

```
                    83     90        100       110       120       130       140       150          164
» Contig 1       (83) TGGAGACAAAGTATGGTACAACATACAACGCTTATCTTATAAAGACCCCAGAAGGTGCAGTTCTCGTCGAAACAGTTAAGGA
» AJ864551.1      (26) TGGAGACAAAGTATGGTACAACATACAACGCTTATCTTATAAAGACCCCAGAAGGTGCAGTTCTCGTCGAAACAGTTAAGGA
» XM_001315387.1 (83) TGGAGACAAAGTATGGTACAACATACAACGCTTATCTTATAAAGACCCCAGAAGGTGCAGTTCTCGTCGAAACAGTTAAGGA
Contig 1         (83) TGGAGACAAAGTATGGTACAACATACAACGCTTATCTTATAAAGACCCCAGAAGGTGCAGTTCTCGTCGAAACAGTTAAGGA
```

Section 3

```
                     165 170       180       190       200       210       220       230             246
» Contig 1       (165) GACATTCTTCGATGAATACATCGAGAAGGTTAAGTCCGTCATCGGCGACATCCACAAGATCAAATACCTTATCACAAACCAT
» AJ864551.1      (108) GACATTCTTCGATGAATACATCGAGAAGGTTAAGTCCGTCATCGGCGACATCCACAAGATCAAATACCTTATCACAAACCAT
» XM_001315387.1 (165) GACATTCTTCGATGAATACATCGAGAAGGTTAAGTCCGTCATCGGCGACATCCACAAGATCAAATACCTTATCACAAACCAT
Contig 1         (165) GACATTCTTCGATGAATACATCGAGAAGGTTAAGTCCGTCATCGGCGACATCCACAAGATCAAATACCTTATCACAAACCAT
```

Section 4

```
                     247          260       270       280       290       300       310                328
» Contig 1       (247) ACAGAACCAGATCACTCTGGCTCCATCAAGAAGATGATCGAGCTCATCCCAGATCTCACAGTCGTTGGCTCCAAGACAGCCC
» AJ864551.1      (190) ACAGAACCAGATCACTCTGGCTCCATCAAGAAGATGATCGAGCTCATCCCAGATCTCACAGTCGTTGGCTCCAAGACAGCCC
» XM_001315387.1 (247) ACAGAACCAGATCACTCTGGCTCCATCAAGAAGATGATCGAGCTCATCCCAGATCTCACAGTCGTTGGCTCCAAGACAGCCC
Contig 1         (247) ACAGAACCAGATCACTCTGGCTCCATCAAGAAGATGATCGAGCTCATCCCAGATCTCACAGTCGTTGGCTCCAAGACAGCCC
```

Section 5

```
                     329        340       350       360       370       380       390       400       410
» Contig 1       (329) TCACATACCTTGAGGATATCGTCAACATCCCATTCAAGGGCCACTCCGCTGAAGATCTCAAGGTCCTTAAGTTCGGTGGCAA
» AJ864551.1      (272) TCACATACCTTGAGGATATCGTCAACATCCCATTCAAGGGCCACTCCGCTGAAGATCTCAAGGTCCTTAAGTTCGGTGGCAA
» XM_001315387.1 (329) TCACATACCTTGAGGATATCGTCAACATCCCATTCAAGGGCCACTCCGCTGAAGATCTCAAGGTCCTTAAGTTCGGTGGCAA
Contig 1         (329) TCACATACCTTGAGGATATCGTCAACATCCCATTCAAGGGCCACTCCGCTGAAGATCTCAAGGTCCTTAAGTTCGGTGGCAA
```


Section 6

```
                     411      420       430       440       450       460       470       480         492
» Contig 1       (411) GTCATTCGAATTCATTTCCTGCCCATTCCTTCACTGGCCAGACTCAATGTACACATGGGTTCCAGAAGAGAAAACACTCTTC
» AJ864551.1      (354) GTCATTCGAATTCATTTCCTGCCCATTCCTTCACTGGCCAGACTCAATGTACACATGGGTTCCAGAAGAGAAGACACTCTTC
» XM_001315387.1 (411) GTCATTCGAATTCATTTCCTGCCCATTCCTTCACTGGCCAGACTCAATGTACACATGGGTTCCAGAAGAGAAGACACTCTTC
Contig 1         (411) GTCATTCGAATTCATTTCCTGCCCATTCCTTCACTGGCCAGACTCAATGTACACATGGGTTCCAGAAGAGAAGACACTCTTC
                                                                                           +
```

Section 7

```
                     493    500       510       520       530       540       550       560             574
» Contig 1       (493) ACATGCGACTCATTCGGTGCTCACTACTCACCAAAGAAGTCCATCCTCATGAGCCAGCTCCCACCAGAGGAAGAGGAAGGCT
» AJ864551.1      (436) ACATGCGACTCATTCGGTGCTCACTACTCACCAAAGAAGTCCATCCTCATGAGCCAGCTCCCACCAGAGGAAGAGGAAGGCT
» XM_001315387.1 (493) ACATGCGACTCATTCGGTGCTCACTACTCACCAAAGAAGTCCATCCTCATGAGCCAGCTCCCACCAGAGGAAGAGGAAGGCT
Contig 1         (493) ACATGCGACTCATTCGGTGCTCACTACTCACCAAAGAAGTCCATCCTCATGAGCCAGCTCCCACCAGAGGAAGAGGAAGGCT
```

Section 8

```
                     575 580       590       600       610       620       630       640               656
» Contig 1       (575) ACCAAGATGCTCTCCTCTACTATTACACCGCCATTTTCGGCCCATTCAAGGAGTACGTCATCAAGGGAACAGACAAGATCCT
» AJ864551.1      (518) ACCAAGATGCTCTCCTCTACTATTACACCGCCATTTTCGGCCCATTCAAGGAGTACGTCATCAAGGGAACAGACAAGATCCT
» XM_001315387.1 (575) ACCAAGATGCTCTCCTCTACTATTACACCGCCATTTTCGGCCCATTCAAGGAGTACGTCATCAAGGGAACAGACAAGATCCT
Contig 1         (575) ACCAAGATGCTCTCCTCTACTATTACACCGCCATTTTCGGCCCATTCAAGGAGTACGTCATCAAGGGAACAGACAAGATCCT
```

Section 9

```
                     657          670       680       690       700       710       720                738
» Contig 1       (657) CAACCTTGACATCAAGCTCGTCGGTCTTGGCCACGGCCCAGTCCTCGATGCTCGTATCAAGGAGACAATCGATACATACCGC
» AJ864551.1      (600) CAACCTTGACATCAAGCTCGTCGGTCTTGGCCACGGCCCAGTCCTCGATGCTCGTATCAAGGAGACAATCGATACATACCGC
» XM_001315387.1 (657) CAACCTTGACATCAAGCTCGTCGGTCTTGGCCACGGCCCAGTCCTCGATGCTCGTATCAAGGAGACAATCGATACATACCGC
Contig 1         (657) CAACCTTGACATCAAGCTCGTCGGTCTTGGCCACGGCCCAGTCCTCGATGCTCGTATCAAGGAGACAATCGATACATACCGC
```

Section 10

```
                     739        750       760       770       780       790       800       810       820
» Contig 1       (739) AAATGGTCTGCCCCACTCCCAACACACGAAGGCAAGGAAGTCGTCATGGTCTACGCCTCTGCTTACGGCTACACAACAGAGA
» AJ864551.1      (682) AAATGGTCTGCCCCACTCCCAACACACGAAGGCAAGGAAGTCGTCATGGTCTACGCCTCTGCTTACGGCTACACAACAGAGA
» XM_001315387.1 (739) AAATGGTCTGCCCCACTCCCAACACACGAAGGCAAGGAAGTCGTCATGGTCTACGCCTCTGCTTACGGCTACACAACAGAGA
Contig 1         (739) AAATGGTCTGCCCCACTCCCAACACACGAAGGCAAGGAAGTCGTCATGGTCTACGCCTCTGCTTACGGCTACACAACAGAGA
```

```
Section 11
                     821      830       840       850       860       870       880       890      902
» Contig 1      (821) TGGCCGAAGAAATCAAGGCAGGCATCCTCGCTAAGATCCCAGATGCCAAGATCAAGATGTTCAACGTCAACATCCAGAACTA
» AJ864551.1     (764) TGGCCGAAGAAATCAAGGCAGGCATCCTCGCTAAGATCCCAGATGCCAAGATCAAGATGTTCAACGTCAACATCCAGAACTA
» XM_001315387.1 (821) TGGCCGAAGAAATCAAGGCAGGCATCCTCGCTAAGATCCCAGATGCCAAGATCAAGATGTTCAACGTCAACATCCAGAACTA
Contig 1        (821) TGGCCGAAGAAATCAAGGCAGGCATCCTCGCTAAGATCCCAGATGCCAAGATCAAGATGTTCAACGTCAACATCCAGAACTA

Section 12
                     903    910       920       930       940       950       960       970         984
» Contig 1      (903) TGGCGGCCTCAAGGGCGAGATCATGAACGCAATCGCTACAGCTGACGGTGTTCTCCTCGGCACAAACACAATCAACGGCGAC
» AJ864551.1     (846) TGGCGGCCTCAAGGGCGAGATCATGAACGCCATCGCTACAGCTGACGGTGTTCTCCTCGGCACAAACACAATCAACGGCGAC
» XM_001315387.1 (903) TGGCGGCCTCAAGGGCGAGATCATGAACGCCATCGCTACAGCTGACGGTGTTCTCCTCGGCACAAACACAATCAACGGCGAC
Contig 1        (903) TGGCGGCCTCAAGGGCGAGATCATGAACGCCATCGCTACAGCTGACGGTGTTCTCCTCGGCACAAACACAATCAACGGCGAC
                                                    +

Section 13
                     985  990       1000      1010      1020      1030      1040      1050          1066
» Contig 1      (985) GCTGTTCCACCAGTTTGGGATGTTGCCCTCTCCATGAACCCAATCGTCCATGGCGGCAAGATCGTCACAGCATTCGGCTCAT
» AJ864551.1     (928) GCTGTTCCACCAGTTTGGGATGTTGCCCTCTCCATGAACCCAATCGTCCATGGCGGCAAGATCGTCACAGCATTCGGCTCAT
» XM_001315387.1 (985) GCTGTTCCACCAGTTTGGGATGTTGCCCTCTCCATGAACCCAATCGTCCATGGCGGCAAGATCGTCACAGCATTCGGCTCAT
Contig 1        (985) GCTGTTCCACCAGTTTGGGATGTTGCCCTCTCCATGAACCCAATCGTCCATGGCGGCAAGATCGTCACAGCATTCGGCTCAT

Section 14
                     1067        1080      1090      1100      1110      1120      1130            1148
» Contig 1     (1067) ACGGCTGGTCCGGCGAAGGTGTCGACAACATCATTGCCAGATTCGATCAGATCCGTTGCAAGGTTATCGACGGCTGCAAGAT
» AJ864551.1    (1010) ACGGCTGGTCCGGCGAAGGTGTCGACAACATCATTGCCAGATTCGATCAGATCCGTTGCAAGGTTATCGACGGCTGCAAGAT
» XM_001315387.1 (1067) ACGGCTGGTCCGGCGAAGGTGTCGACAACATCATTGCCAGATTCGATCAGATCCGTTGCAAGGTTATCGACGGCTGCAAGAT
Contig 1       (1067) ACGGCTGGTCCGGCGAAGGTGTCGACAACATCATTGCCAGATTCGATCAGATCCGTTGCAAGGTTATCGACGGCTGCAAGAT

Section 15
                     1149       1160      1170      1180      1190      1200      1210      1220    1230
» Contig 1     (1149) CAAGTTCCGCATGTCAAAGAAGGAACACGGGAAGGTCAACGAATTCGGCCAGCAGTTCGGTGAAGCCCTCATCACAGGCAAG
» AJ864551.1    (1092) CAAGTTCCGCATGTCAAAGAAGGAACACGGGAAGGTCAACGAATTCGGCCAGCAGTTCGGTGAAGCCCTCATCACAGGCAAG
» XM_001315387.1 (1149) CAAGTTCCGCATGTCAAAGAAGGAACACGGGAAGGTCAACGAATTCGGCCAGCAGTTCGGTGAAGCCCTCATCACAGGCAAG
Contig 1       (1149) CAAGTTCCGCATGTCAAAGAAGGAACACGGGAAGGTCAACGAATTCGGCCAGCAGTTCGGTGAAGCCCTCATCACAGGCAAG
```



```
Section 16
                     1231      1240      1250      1260      1270      1280      1290      1300     1312
» Contig 1     (1231) GTCCCAGAACGCGCTGTTCCAGGCAAGGTTGTTGGTGCTAAGGACTGGTCCGAGCTCAACCCAACAGGCGCCGTCGTTCTCT
» AJ864551.1    (1174) GTCCCAGAACGCGCTGTTCCAGGCAAGGTTGTTGGTGCTAAGGACTGGTCCGAGCTCAACCCAACAGGCGCCGTCGTTCTCT
» XM_001315387.1 (1231) GTCCCAGAACGCGCTGTTCCAGGCAAGGTTGTTGGTGCTAAGGACTGGTCCGAGCTCAACCCAACAGGCGCCGTCGTTCTCT
Contig 1       (1231) GTCCCAGAACGCGCTGTTCCAGGCAAGGTTGTTGGTGCTAAGGACTGGTCCGAGCTCAACCCAACAGGCGCCGTCGTTCTCT

Section 17
                     1313   1320      1330      1340      1350      1360      1370      1380      1394
» Contig 1     (1313) GGCGTTGCGTCATCTGCGGCGAAATCTACGCCGGTGTTACACCACCACTCCAGTGCCCAGCTTGCGGTGTCGGCCAAGACCT
» AJ864551.1    (1256) GGCGTTGCGTCATCTGCGGCGAAATCTACGCCGGTGTTACACCACCACTCCAGTGCCCAGCTTGCGGTGTCGGCCAAGACCT
» XM_001315387.1 (1313) GGCGTTGCGTCATCTGCGGCGAAATCTACGCCGGTGTTACACCACCACTCCAGTGCCCAGCTTGCGGTGTCGGCCAAGACCT
Contig 1       (1313) GGCGTTGCGTCATCTGCGGCGAAATCTACGCCGGTGTTACACCACCACTCCAGTGCCCAGCTTGCGGTGTCGGCCAAGACCT

Section 18
                     1395 1400      1410      1420      1430      1440      1450      1460          1476
» Contig 1     (1395) CTTCGAACTCTACGAGGCCGAGCAGGTCACACACAAGTCAACAGAGCCACTCAACTACGTCATCGTTGGCTCTGGTGCCGCC
» AJ864551.1    (1338) CTTCGAACTCTACGAGGCCGAGCAGGTCACACACAAGTCAACAGAGCCACTCAACTACGTCATCGTTGGCTCTGGTGCCGCC
» XM_001315387.1 (1395) CTTCGAACTCTACGAGGCCGAGCAGGTCACACACAAGTCAACAGAGCCACTCAACTACGTCATCGTTGGCTCTGGTGCCGCC
Contig 1       (1395) CTTCGAACTCTACGAGGCCGAGCAGGTCACACACAAGTCAACAGAGCCACTCAACTACGTCATCGTTGGCTCTGGTGCCGCC

Section 19
                     1477        1490      1500      1510      1520      1530      1540             1558
» Contig 1     (1477) GCTGTTGCTTCCATCGAAGCCATCCGCGCTCGCAATGCCGTCGCTAAGATCACAATGATCAGC-GTGAAAAGGTCATGCCAT
» AJ864551.1    (1420) GCTGTTGCTTCCATCGAAGCCATCCGCGCGCGCAATGCCGTCGCTAAGATCACAATGATCAGCCGTGAAAAGGTCATGCCAT
» XM_001315387.1 (1477) GCTGTTGCTTCCATCGAAGCCATCCGCGCGCGCAATGCCGTCGCTAAGATCACAATGATCAGCCGTGAAAAGGTCATGCCAT
Contig 1       (1477) GCTGTTGCTTCCATCGAAGCCATCCGCGCGCGCAATGCCGTCGCTAAGATCACAATGATCAGCCGTGAAAAGGTCATGCCAT
                                                   +                                  +

Section 20
                     1559       1570      1580      1590      1600      1610      1620      1630    1640
» Contig 1     (1559) ACTACAGACCAATCATCGTCGATGCCCTCAACGCTGAGATCGCTCCAGACAAATACTTCCTCAAGAACGAAGAGTGGTACAA
» AJ864551.1    (1502) ACTACAGACCAATCATCGTCGATGCCCTCAACGCTGAGATCGCTCCAGACAAATACTTCCTCAAGAACGAAGAGTGGTACAA
» XM_001315387.1 (1559) ACTACAGACCAATCATCGTCGATGCCCTCAACGCTGAGATCGCTCCAGACAAATACTTCCTCAAGAACGAAGAGTGGTACAA
Contig 1       (1559) ACTACAGACCAATCATCGTCGATGCCCTCAACGCTGAGATCGCTCCAGACAAATACTTCCTCAAGAACGAAGAGTGGTACAA
```

Section 21

```
                     1641      1650      1660      1670      1680      1690      1700      1710      1722
» Contig 1 (1641)    GGAGAACAAGATCGAAATCAAGCTTGACACATCCGTCACAGCCATCGACACAAAGGCCAAGAAGGTCAAGCTCTGCTGCGGT
» AJ864551.1 (1584)   GGAGAACAAGATCGAAATCAAGCTTGACACATCCGTCACAGCCATCGACACAAAGGCCAAGAAGGTCAAGCTCTGCCGCGGT
» XM_001315387.1 (1641) GGAGAACAAGATCGAAATCAAGCTTGACACATCCGTCACAGCCATCGACACAAAGGCCAAGAAGGTCAAGCTCTGCCGCGGT
Contig 1 (1641)      GGAGAACAAGATCGAAATCAAGCTTGACACATCCGTCACAGCCATCGACACAAAGGCCAAGAAGGTCAAGCTCTGCCGCGGT
                                                                                                     +
```

Section 22

```
                     1723      1730      1740      1750      1760      1770      1780      1790      1804
» Contig 1 (1723)    GATGAACTCGCCTACGACAAGCTCATCCTCGCTACAGGTTCCCGCCCATTCATCCCACCAATCGGCCACGAGGGACTCTCCG
» AJ864551.1 (1666)   GATGAACTCGCCTACGACAAGCTCATCCTCGCTACAGGTTCCCGCCCATTCATCCCACCAATCGGCCACGAGGGACTCTCCG
» XM_001315387.1 (1723) GATGAACTCGCCTACGACAAGCTCATCCTCGCTACAGGTTCCCGCCCATTCATCCCACCAATCGGCCACGAGGGACTCTCCG
Contig 1 (1723)      GATGAACTCGCCTACGACAAGCTCATCCTCGCTACAGGTTCCCGCCCATTCATCCCACCAATCGGCCACGAGGGACTCTCCG
```

Section 23

```
                     1805      1810      1820      1830      1840      1850      1860      1870      1886
» Contig 1 (1805)    GCGTCATCGTCATCCGTACATCCGCTGATGTCGACCAGCTCAAGGCCGCCTGCCAGACAGCTAAGAAGGCCGTCGTTATCGG
» AJ864551.1 (1748)   GCGTCATCGTCATCCGTACATCCGCTGATGTCGACCAGCTCAAGGCCGCCTGCCAGACAGCTAAGAAGGCCGTCGTTATCGG
» XM_001315387.1 (1805) GCGTCATCGTCATCCGTACATCCGCTGATGTCGACCAGCTCAAGGCCGCCTGCCAGACAGCTAAGAAGGCCGTCGTTATCGG
Contig 1 (1805)      GCGTCATCGTCATCCGTACATCCGCTGATGTCGACCAGCTCAAGGCCGCCTGCCAGACAGCTAAGAAGGCCGTCGTTATCGG
```

Section 24

```
                     1887      1900      1910      1920      1930      1940      1950      1968
» Contig 1 (1887)    TGGTGGTGTCCTCGGTCTCGAGAACGCATCCGCTATCAAGGAGAAGGGCGTTGCTGTCACAGTCGTCGAGTGCATGCCACGT
» AJ864551.1 (1830)   TGGTGGTGTCCTCGGTCTCGAGAACGCATCCGCTATCAAGGAGAAGGGCGTTGCTGTCACAGTCGTCGAGTGCATGCCACGT
» XM_001315387.1 (1887) TGGTGGTGTCCTCGGTCTCGAGAACGCATCCGCTATCAAGGAGAAGGGCGTTGCTGTCACAGTCGTCGAGTGCATGCCACGT
Contig 1 (1887)      TGGTGGTGTCCTCGGTCTCGAGAACGCATCCGCTATCAAGGAGAAGGGCGTTGCTGTCACAGTCGTCGAGTGCATGCCACGT
```

Section 25

```
                     1969      1980      1990      2000      2010      2020      2030      2040      2050
» Contig 1 (1969)    CTCATGGCCCGCCAGCTCGATACAGAAGCTTCCGCTGTCCTCCTCGAGGAAGTCAAGAAGTTTGGTGTCGATGTCCGCCTCG
» AJ864551.1 (1912)   CTCATGGCCCGCCAGCTCGATACAGAAGCTTCCGCTGTCCTCCTCGAGGAAGTCAAGAAGTTTGGTGTCGATGTCCGCCTCG
» XM_001315387.1 (1969) CTCATGGCCCGCCAGCTCGATACAGAAGCTTCCGCTGTCCTCCTCGAGGAAGTCAAGAAGTTTGGTGTCGATGTCCGCCTCG
Contig 1 (1969)      CTCATGGCCCGCCAGCTCGATACAGAAGCTTCCGCTGTCCTCCTCGAGGAAGTCAAGAAGTTTGGTGTCGATGTCCGCCTCG
```


Section 26

```
                     2051      2060      2070      2080      2090      2100      2110      2120      2132
» Contig 1 (2051)    GCATGACAGTTTCCATCAAGGGCGATGGCAAGCACGTCACAGGCGTCCAGGTCGGCGAGGAGTTCATCCCAGCCGATTTCGT
» AJ864551.1 (1994)   GCATGACAGTTTCCATCAAGGGCGATGGCAAGCACGTCACAGGCGTCCAGGTCGGCGAGGAGTTCATCCCAGCCGATTTCGT
» XM_001315387.1 (2051) GCATGACAGTTTCCATCAAGGGCGATGGCAAGCACGTCACAGGCGTCCAGGTCGGCGAGGAGTTCATCCCAGCCGATTTCGT
Contig 1 (2051)      GCATGACAGTTTCCATCAAGGGCGATGGCAAGCACGTCACAGGCGTCCAGGTCGGCGAGGAGTTCATCCCAGCCGATTTCGT
```

Section 27

```
                     2133      2140      2150      2160      2170      2180      2190      2200      2214
» Contig 1 (2133)    TGTTGTCAACGCCGGTGTCCGCGCCGAATCCGAAGTAGCCGCTGCAGCTGGCATCAAGTGCGGCCGCGGTATCATTGTCAAC
» AJ864551.1 (2076)   TGTTGTCAACGCCGGTGTCCGCGCCGAATCCGAAGTAGCCGCTGCAGCTGGCATCAAGTGCGGCCGCGGTATCATTGTCAAC
» XM_001315387.1 (2133) TGTTGTCAACGCCGGTGTCCGCGCCGAATCCGAAGTAGCCGCTGCAGCTGGCATCAAGTGCGGCCGCGGTATCATTGTCAAC
Contig 1 (2133)      TGTTGTCAACGCCGGTGTCCGCGCCGAATCCGAAGTAGCCGCTGCAGCTGGCATCAAGTGCGGCCGCGGTATCATTGTCAAC
```

Section 28

```
                     2215      2220      2230      2240      2250      2260      2270      2280      2296
» Contig 1 (2215)    AACAAGATGGAGACAAACGTTCCAGATGTCTACGCAGCTGGTGATTGCGCATTCCTTGACAACATGAACCAGGGTCTCTGGG
» AJ864551.1 (2158)   AACAAGATGGAGACAAACGTTCCAGATGTCTACGCAGCTGGTGATTGCGCATTCCTTGACAACATGAACCAGGGTCTCTGGG
» XM_001315387.1 (2215) AACAAGATGGAGACAAACGTTCCAGATGTCTACGCAGCTGGTGATTGCGCATTCCTTGACAACATGAACCAGGGTCTCTGGG
Contig 1 (2215)      AACAAGATGGAGACAAACGTTCCAGATGTCTACGCAGCTGGTGATTGCGCATTCCTTGACAACATGAACCAGGGTCTCTGGG
```

Section 29

```
                     2297      2310      2320      2330      2340      2350      2360      2378
» Contig 1 (2297)    CTCCAGCTCTTGCTATGGGTAAGGTCGCCGGTGCTAACGCTTGCGGCGATAACAAGACATTCGCTTTCGCTATCGAACCAGT
» AJ864551.1 (2240)   CTCCAGCTCTTGCTATGGGTAAGGTCGCCGGTGCTAACGCTTGCGGCGATAACAAGACATTCGCTTTCGCTATCGAACCAGT
» XM_001315387.1 (2297) CTCCAGCTCTTGCTATGGGTAAGGTCGCCGGTGCTAACGCTTGCGGCGATAACAAGACATTCGCTTTCGCTATCGAACCAGT
Contig 1 (2297)      CTCCAGCTCTTGCTATGGGTAAGGTCGCCGGTGCTAACGCTTGCGGCGATAACAAGACATTCGCTTTCGCTATCGAACCAGT
```

Section 30

```
                     2379      2390      2400      2410      2420      2430      2440      2450      2460
» Contig 1 (2379)    CTCAATGATCGCTATGGGAACAGATCTCTTCGCTTGCGGCAACCCACCAGAGAGCGCACAGGGCTACAACGTTGTCTCTCAG
» AJ864551.1 (2322)   CTCAATGATCGCTATGGGAACAGATCTCTTCGCTTGCGGCAACCCACCAGAGAGCGCACAGGGCTACAACGTTGTCTCTCAG
» XM_001315387.1 (2379) CTCAATGATCGCTATGGGAACAGATCTCTTCGCTTGCGGCAACCCACCAGAGAGCGCACAGGGCTACAACGTTGTCTCTCAG
Contig 1 (2379)      CTCAATGATCGCTATGGGAACAGATCTCTTCGCTTGCGGCAACCCACCAGAGAGCGCACAGGGCTACAACGTTGTCTCTCAG
```

```
                                                                                   Section 31
                       2461                                                             2542
   » Contig 1 (2461)   AAGGATGACAAGGAAGGATCCGCTATCAAGCTCTACTTCAAGGATAATCGCCTCGTTTACGCAGCTGGCTTCAGAATCCAGA
  » AJ864551.1 (2404)  AAGGATGACAAGGAAGGATCCGCTATCAAGCTCTACTTCAAGGATAATCGCCTCGTTTACGCAGCTGGCTTCAGAATCCAGA
» XM_001315387.1 (2461) AAGGATGACAAGGAAGGATCCGCTATCAAGCTCTACTTCAAGGATAATCGCCTCGTTTACGCAGCTGGCTTCAGAATCCAGA
     Contig 1 (2461)   AAGGATGACAAGGAAGGATCCGCTATCAAGCTCTACTTCAAGGATAATCGCCTCGTTTACGCAGCTGGCTTCAGAATCCAGA

                                                                                   Section 32
                       2543                                                             2624
   » Contig 1 (2543)   AGATCTCCGGCTCCCTCTTGAAGGGCGTCAGAGAGGGCCGCTCATTAGAGCGCATCATGGCTGAAATCTTCTAAAGAAGATT
  » AJ864551.1 (2486)  AGATCTCCGGCTCCCTCTTGAAGGGCGTCAGAGAGGGCCGCTCATTAGAGCGCATCATGGCTGAAATCTTCTAAAGAAGATT
» XM_001315387.1 (2543) AGATCTCCGGCTCCCTCTTGAAGGGCGTCAGAGAGGGCCGCTCATTAGAGCGCATCATGGCTGAAATCTTCTAAAGAAGATT
     Contig 1 (2543)   AGATCTCCGGCTCCCTCTTGAAGGGCGTCAGAGAGGGCCGCTCATTAGAGCGCATCATGGCTGAAATCTTCTAAAGAAGATT

                                                                                   Section 33
                       2625                                                             2706
   » Contig 1 (2625)   TCCAAAAAATAATTTTAATAATTTCGATGTTCGAAACGGCTCGCTTTGGTAAAAAAAGTTTTAATAATCTTCTCAATTTTTT
  » AJ864551.1 (2568)  TCCAAAAAATAATTTTAATAATTTCGATGTTCGAAACGGCTCGCTTTGGTAAAAAAAGTTTTAATAATCTTCTCAATTTTTT
» XM_001315387.1 (2625) TCCAAAAAATAATTTTAATAATTTCGATGTTCGAAACGGCTCGCTTTGGTAAAAAAAGTTTTAATAATCTTCTCAA
     Contig 1 (2625)   TCCAAAAAATAATTTTAATAATTTCGATGTTCGAAACGGCTCGCTTTGGTAAAAAAAGTTTTAATAATCTTCTCAATTTTTT

                                                                                   Section 34
                       2707                                                             2788
   » Contig 1 (2707)   TCCAAATATTTTTTTCCATTGAAAAGAATTTTAAAATTGAATTAATAAAACAGTTTTCTTGATATAAATTTTGACATTCATT
  » AJ864551.1 (2650)  TCCAAATATTTTTTTCCATTGAAAAGAATTTTAAAATTGAATTAATAAAACAGTTTTCTTGATATAAA
» XM_001315387.1(•2699)
     Contig 1 (2707)   TCCAAATATTTTTTTCCATTGAAAAGAATTTTAAAATTGAATTAATAAAACAGTTTTCTTGATATAAATTTTGACATTCATT

                                                                                   Section 35
                       2789                                         2835
   » Contig 1 (2789)   TTTCAAAAATGGAAGAGTTTATTGAGCGTTTAAAGTCAATTTATGAG
  » AJ864551.1(•2716)
» XM_001315387.1(•2699)
     Contig 1 (2789)   TTTCAAAAATGGAAGAGTTTATTGAGCGTTTAAAGTCAATTTATGAG
```

Protein in C1 :

Red : missense

MLKIQQLTEDIYWLGVLDSNLAVFDIIMETKYGTTYNAYLIKTPEGAVLVETVKETFFDEYIEKVKSVIGDIHKIKYLITN
HTEPDHSGSIKKMIELIPDLTVVGSKTALTYLEDIVNIPFKGHSAEDLKVLKFGGKSFEFISCPFLHWPDSMYTWVPEE
KTLFTCDSFGAHYSPKKSILMSQLPPEEEEGYQDALLYYYTAIFGPFKEYVIKGTDKILNLDIKLVGLGHGPVLDARIKET
IDTYRKWSAPLPTHEGKEVVMVYASAYGYTTEMAEEIKAGILAKIPDAKIKMFNVNIQNYGGLKGEIMNAIATADG
VLLGTNTINGDAVPPVWDVALSMNPIVHGGKIVTAFGSYGWSGEGVDNIIARFDQIRCKVIDGCKIKFRMSKKEHG
KVNEFGQQFGEALITGKVPERAVPGKVVGAKDWSELNPTGAVVLWRCVICGEIYAGVTPPLQCPACGVGQDLFEL
YEAEQVTHKSTEPLNYVIVGSGAAAVASIEAIRARNAVAKITMISVKRSCHTTDQSSSMPSTLRSLQTNTSSRTKSGTR
RTRSKSSLTHPSQPSTQRPRRSSSAAVMNSPTTSSSSLQVPAHSSHQSATRDSPASSSSVHPLMSTSSRPPARQLRRP
SLSVVVSSVSRTHPLSRRRALLSQSSSACHVSWPASSIQKLPLSSSRKSRSLVSMSASA

XP_001315422

MLKIQQLTEDIYWLGVLDSNLAVFDIIMETKYGTTYNAYLIKTPEGAVLVETVKETFFDEYIEKVKSVIGDIHKIKYLITN
HTEPDHSGSIKKMIELIPDLTVVGSKTALTYLEDIVNIPFKGHSAEDLKVLKFGGKSFEFISCPFLHWPDSMYTWVPEE
KTLFTCDSFGAHYSPKKSILMSQLPPEEEEGYQDALLYYYTAIFGPFKEYVIKGTDKILNLDIKLVGLGHGPVLDARIKET
IDTYRKWSAPLPTHEGKEVVMVYASAYGYTTEMAEEIKAGILAKIPDAKIKMFNVNIQNYGGLKGEIMNAIATADG
VLLGTNTINGDAVPPVWDVALSMNPIVHGGKIVTAFGSYGWSGEGVDNIIARFDQIRCKVIDGCKIKFRMSKKEHG
KVNEFGQQFGEALITGKVPERAVPGKVVGAKDWSELNPTGAVVLWRCVICGEIYAGVTPPLQCPACGVGQDLFEL
YEAEQVTHKSTEPLNYVIVGSGAAAVASIEAIRARNAVAKITMISREKVMPYYRPIIVDALNAEIAPDKYFLKNEEWYK
ENKIEIKLDTSVTAIDTKAKKVKLCRGDELAYDKLILATGSRPFIPPIGHEGLSGVIVIRTSADVDQLKAACQTAKKAVVI
GGGVLGLENASAIKEKGVAVTVVECMPRLMARQLDTEASAVLLEEVKKFGVDVRLGMTVSIKGDGKHVTGVQVG
EEFIPADFVVVNAGVRAESEVAAAAGIKCGRGIIVNNKMETNVPDVYAAGDCAFLDNMNQGLWAPALAMGKVA
GANACGDNKTFAFAIEPVSMIAMGTDLFACGNPPESAQGYNVVSQKDDKEGSAIKLYFKDNRLVYAAGFRIQKISG
SLLKGVREGRSLERIMAEIF

Protein C1 without mutation: = 100% identical to XP_001315422

MLKIQQLTEDIYWLGVLDSNLAVFDIIMETKYGTTYNAYLIKTPEGAVLVETVKETFFDEYIEKVKSVIGDIHKIKYLITN
HTEPDHSGSIKKMIELIPDLTVVGSKTALTYLEDIVNIPFKGHSAEDLKVLKFGGKSFEFISCPFLHWPDSMYTWVPEE
KTLFTCDSFGAHYSPKKSILMSQLPPEEEEGYQDALLYYYTAIFGPFKEYVIKGTDKILNLDIKLVGLGHGPVLDARIKET
IDTYRKWSAPLPTHEGKEVVMVYASAYGYTTEMAEEIKAGILAKIPDAKIKMFNVNIQNYGGLKGEIMNAIATADG
VLLGTNTINGDAVPPVWDVALSMNPIVHGGKIVTAFGSYGWSGEGVDNIIARFDQIRCKVIDGCKIKFRMSKKEHG
KVNEFGQQFGEALITGKVPERAVPGKVVGAKDWSELNPTGAVVLWRCVICGEIYAGVTPPLQCPACGVGQDLFEL
YEAEQVTHKSTEPLNYVIVGSGAAAVASIEAIRARNAVAKITMISREKVMPYYRPIIVDALNAEIAPDKYFLKNEEWYK
ENKIEIKLDTSVTAIDTKAKKVKLCCGDELAYDKLILATGSRPFIPPIGHEGLSGVIVIRTSADVDQLKAACQTAKKAVVI
GGGVLGLENASAIKEKGVAVTVVECMPRLMARQLDTEASAVLLEEVKKFGVDVRLGMTVSIKGDGKHVTGVQVG
EEFIPADFVVVNAGVRAESEVAAAAGIKCGRGIIVNNKMETNVPDVYAAGDCAFLDNMNQGLWAPALAMGKVA
GANACGDNKTFAFAIEPVSMIAMGTDLFACGNPPESAQGYNVVSQKDDKEGSAIKLYFKDNRLVYAAGFRIQKISG
SLLKGVREGRSLERIMAEIF
